# Stability and performance analysis of storage root yield in a dataset of sweet potato varieties (*Ipomoea batatas* L.)

**DOI:** 10.1016/j.dib.2024.110493

**Published:** 2024-05-08

**Authors:** Zakaria Alam, Sanjida Akter, Md. Anwar Hossain Khan, Md. Shamshul Alam, Md. Nazmul Islam, Abul Fazal Mohammad Shamim Ahsan, Mohammad Saidur Rahman, Md. Babul Anwar, Mousumi Sultana, S.M. Kamrul Hasan Chowdhury, Mohammad Quamrul Islam Matin, A.K.M. Zonayed-Ull-Noor, Mohammad Mainuddin Molla, Umakanta Sarker

**Affiliations:** a Bangladesh Agricultural Research Institute, Gazipur-1701, Bangladesh; b Bangladesh Rice research Institute, Bangladesh; c Bangabandhu Sheikh Mujibur Rahman Agricultural University, Bangladesh

**Keywords:** Genotype by environment interaction (GEI), Genetic parameter, AMMI analysis, WAAS biplot, GGE biplot

## Abstract

The dataset focuses on evaluating the performance of 17 sweet potato varieties (G) released by the Bangladesh Agricultural Research Institute (BARI) in terms of storage root yield and stability across five locations (E) in Bangladesh—Gazipur, Bogura, Jamalpur, Jashore, and Chattogram. The result revealed that BARI Mistialu-12 exhibited the highest average storage root yield at 45.35 t/ha, closely followed by BARI Mistialu-16 at 44.64 t/ha. Conversely, BARI Mistialu-1 had the lowest mean yield of 25.99 t/ha. Among the locations, Bogura recorded the highest mean root yield at 37.05 t/ha, while Chattogram exhibited the lowest at 31.27 t/ha. A combined analysis of variance revealed the presence of variability in storage root yield attributed to the genotype-location (environment) interaction (GEI). To delve deeper into this interaction, additive and multiplicative interaction effect models (AMMI) along with a linear mixed model (LMM) were employed for further investigations to confirm the significant contribution of GEI variance to root yield. The LMM results showed genetic variance (%), heritability (%), selection accuracy (%), and GEI correlation coefficients of 52.27, 54, 94, and 30, respectively. The AMMI analysis indicated that the first two principal components accounted for 74.60 % of GEI, with 20.16 % attributed to it. Assessing significant Interaction Principal Component Analyses (IPCAs) through the Weighted Average of Absolute Scores (WAAS) indicated that BARI Mistialu-12 is the most stable genotype, followed by BARI Mistialu-16 and BARI Mistialu-8, all displaying above-average root yield. The mega-environment analysis associated the highest root production of BARI Mistialu-11 and BARI Mistialu-2 with the Jamalpur location, while Gazipur, Bogura, and Jashore were linked with the superior performance of BARI Mistialu-12 and BARI Mistialu-16 genotypes. These findings are crucial for future breeding programs and the rapidly growing sweet potato industry, given the stable high-yield potential across diverse agro-ecological conditions. However, it is imperative to repeat the study to ensure reliable outcomes.

Specifications Table

**Table:** 

Subject	Agricultural and Biological Science
Specific subject area	Agronomy and Crop Science
Data format	Raw
Type of data	Table and Figures
How the data were collected	Harvesting was done 130 days after vine planting, with a random selection of 10 plants from each plot for every replication. The analytical balance was employed for weighing the storage roots of sweet potato.
Data source location	The present study was conducted during 2022–23 growing season at five locations in Bangladesh: Gazipur, Bogura, Jamalpur, Chattogram, and Jashore. The study area lies within Bangladesh's geographic coordinates, ranging from 23.6850° N latitude to 90.3563° E longitude. It spans elevations from 10 m (Coastal South) to 105 m (North) above sea level.
Data accessibility	https://data.mendeley.com/datasets/g4jpd7r2zf/1
Related research article	Research article has been submitted to journal

## 1 Value of the Data

• The dataset explores the performance of different sweet potato varieties in terms of storage root yield, offering valuable insights for both the sweet potato industry and small-scale farmers.
• The dataset connects genotypes to particular environments, underscoring the significance of choosing varieties based on their appropriateness for achieving optimal root yield. This information offers valuable insights for sweet potato breeders, the industry, and small-scale farmers.
• The dataset's variance analysis showed root yield variability due to genotype by location (environment) interaction (GEI). Subsequent AMMI and LMM investigations confirmed significant GEI variance. The LMM supplied genetic variance, heritability, selection accuracy, and GEI correlation coefficients, aiding insights breeders for selecting superior sweet potato genotypes.

## 2 Background

There is an urgent need to identify sweet potato varieties in Bangladesh that not only demonstrate high yield potential but also exhibit stability across diverse environmental conditions. Currently, local farmers are cultivating sweet potatoes with an average yield of approximately 10.50 tons per hectare [[1], [2], [3], [4], [5]], showing poor stability [2,3]. Some of the 17 sweet potato varieties developed by the Bangladesh Agricultural Research Institute (BARI) are experiencing reduced root yields in specific agro-ecologies of the country [[2], [3], [4], [5]]. Given this situation, it is crucial for plant breeders to evaluate yield performance and ensure consistent phenotypic expression by selecting genotypes (G) that demonstrate stability or adaptability to particular environments (E), minimizing genotype by environment interaction (GEI). Limited research has been conducted on the stability of released sweet potato varieties in Bangladesh, prompting sweet potato breeders to identify high-yielding and stable varieties for inclusion in multi-environment trials (METs).

## 3 Data Description

The dataset presented in this article includes two figures and four tables. In ANOVA, a significant level of variation (*p* < 0.001) was observed in sweet potato root yield among the different varieties/genotypes (G), locations (E), and the genotype-location (environment) interaction (GEI, as indicated in Table 1. The mean sum of squares for residuals was the smallest at 7.9, followed by GEI at 47.8, locations at 262.4, and genotypes at 410.4. The variation percentages for G, E, and GEI were 52.27, 33.42, and 6.09, respectively. The coefficient of variation for the tested sweet potato genotypes was relatively low at 8.14. The significance of G and GEI on root yield was further confirmed at a p-value of less than 0.001 through the likelihood ratio test (LRT) (Table 1). In the random effect model, the findings indicated a relatively low heritability value of 54 % for root yield. The correlation coefficient for GEI was modest, standing at 0.30. Additionally, the selection accuracy was high, reaching 0.94.

**Table 1:** A combined ANOVA and estimation of genetic parameter using LMM of storage root yield for 17 BARI-released sweet potato varieties studied across five locations with a randomized complete block (RCB) design during 2022–23 growing season.

SV	DF	SS	MSS^s^	Variance (%)	LRT^s^	r^2^	h^2^	SE	CV
E	4	1049	262.4^⁎⁎⁎^	33.42	–	–	–	0.94	8.14
R	2	113	56.6^⁎⁎⁎^	7.21	–	–	–		
G	16	6567	410.4^⁎⁎⁎^	52.27	39.44^⁎⁎⁎^	–	0.54		
GEI	64	3061	47.8^⁎⁎⁎^	6.09	89.82^⁎⁎⁎^	0.30	–		
Residuals	168	1332	7.9	1.01	–	–	–		

SV = source of variation, *E* = location, *G* = genotype, *R* = replication, GEI= genotype-location interaction, DF = degrees of freedom, SS = sum of squares, MSS = mean sum of squares, LRT= likelihood ratio test (LRT), r^2^= GEI correlation of coefficient, h^2^= heritability, SE= selection accuracy, CV = coefficient of variations.

s Significant at *p* ≤ 0.001.

Table 2 displays the average performance pattern of root yield for 17 sweet potato varieties across five locations. E2 registered the highest root yield at 37.05 t/ha, statistically comparable to E3 at 36.15 t/ha, followed by E1 at 34.70 t/ha, E4 at 33.55 t/ha, and E5 at 31.27 t/ha. Among the genotypes, G12 recorded the highest root yield at 45.35 t/ha, statistically similar to G16 at 44.64 t/ha, while the lowest yields were observed in G1 at 25.99 t/ha and G17 at 27.51 t/ha. Regarding the interaction effect, the highest mean yield was noted in the E1 location with G12 at 55.08 t/ha, whereas the lowest yield was in the E3 location with G1 at 24.33 t/ha.

**Table 2:** The mean storage root yield of 17 BARI-released sweet potato varieties across five locations in Bangladesh during 2022–23 growing season.

Genotypic code	Storage root yield (t/ha)					GM^R^
	Locational code					
	E1	E2	E3	E4	E5	
G1	26.00	26.74	24.33	24.63	28.25	25.99^17^
G2	42.82	32.78	44.86	30.74	29.59	36.16^5^
G3	26.67	37.78	29.79	31.22	30.21	31.14^14^
G4	30.57	31.67	30.68	33.93	28.59	31.09^15^
G5	31.67	32.21	37.05	35.26	29.16	33.07^10^
G6	31.37	39.44	31.34	33.07	31.18	33.28^9^
G7	32.00	35.88	36.88	32.33	29.77	33.37^8^
G8	41.15	44.85	35.84	37.30	35.88	39.00^4^
G9	31.44	37.98	29.25	34.07	28.78	32.31^12^
G10	31.56	37.60	34.86	32.41	27.18	32.72^11^
G11	34.00	45.16	49.74	32.00	37.67	39.71^3^
G12	51.85	47.64	48.19	42.18	36.90	45.35^1^
G13	33.70	38.40	47.14	32.96	28.55	36.15^6^
G14	33.30	35.36	32.68	28.34	29.63	31.86^13^
G15	42.59	34.58	29.71	29.89	32.74	33.90^7^
G16	42.15	46.86	45.31	47.78	41.09	44.64^2^
G17	27.02	24.99	26.85	32.22	26.46	27.51^16^
EM^R^	34.70^3^	37.05^1^	36.15^2^	33.55^4^	31.27^5^	

GM = genotypic mean, EM = Locational mean.

R rank based on mean storage root yield performance (high to low mean).

Table 3 displays the AMMI analysis of variance for the root yield of 17 sweet potato genotypes assessed across five locations in Bangladesh. The analysis revealed that the root yield performance was significantly impacted by E, G, and GEI, with a p-value below 0.001. The contributions to variance from E and G were 6.91 % and 43.25 % of the total sum of squares, respectively, while GEI accounted for 47.82 % of the overall variation. GEI constituted the majority of the total sum of squares in the model. The cumulative variation from E, G, and GEI collectively, referred to as treatments, contributed more to root yield (70.32 %) than the error (7.91 %).

**Table 3:** ANOVA of the AMMI for the root yield of 17 sweet potato genotypes studied across five locations of Bangladesh during 2022–23 growing season.

SV	DF	SS	MSS^s^	TSS (%)	GEI (%)	GEIC (%)
T	84	7616.52	90.67^⁎⁎⁎^	70.32	–	–
E	4	1049.41	262.35^⁎⁎⁎^	6.91	–	–
R(E)	10	244.65	24.47^⁎⁎⁎^	1.61	–	–
G	16	6567.11	410.44^⁎⁎⁎^	43.25	–	–
GEI	64	3060.52	47.82^⁎⁎⁎^	20.16	–	–
PC1	19	1347.95	70.94^⁎⁎⁎^	8.88	44	44
PC2	17	934.44	54.97^⁎⁎⁎^	6.15	30.5	74.6
Residuals	28	778.12	27.79^⁎⁎⁎^	5.12	25.4	100
Error	160	1200.90	7.51	7.91	–	–
Total	318	15,183.10	47.75	–	–	–

SV = source of variation, E= location, R = replication, G= genotype, T = treatments (G+E+GEI), GEIC= GEI cumulative of PCs, PC= principal component, DF = degrees of freedom, SS = sum of squares, TSS = total sum of squares, MSS = mean sum of squares.

s Significant at *p* ≤ 0.001.

**Fig. 1:**
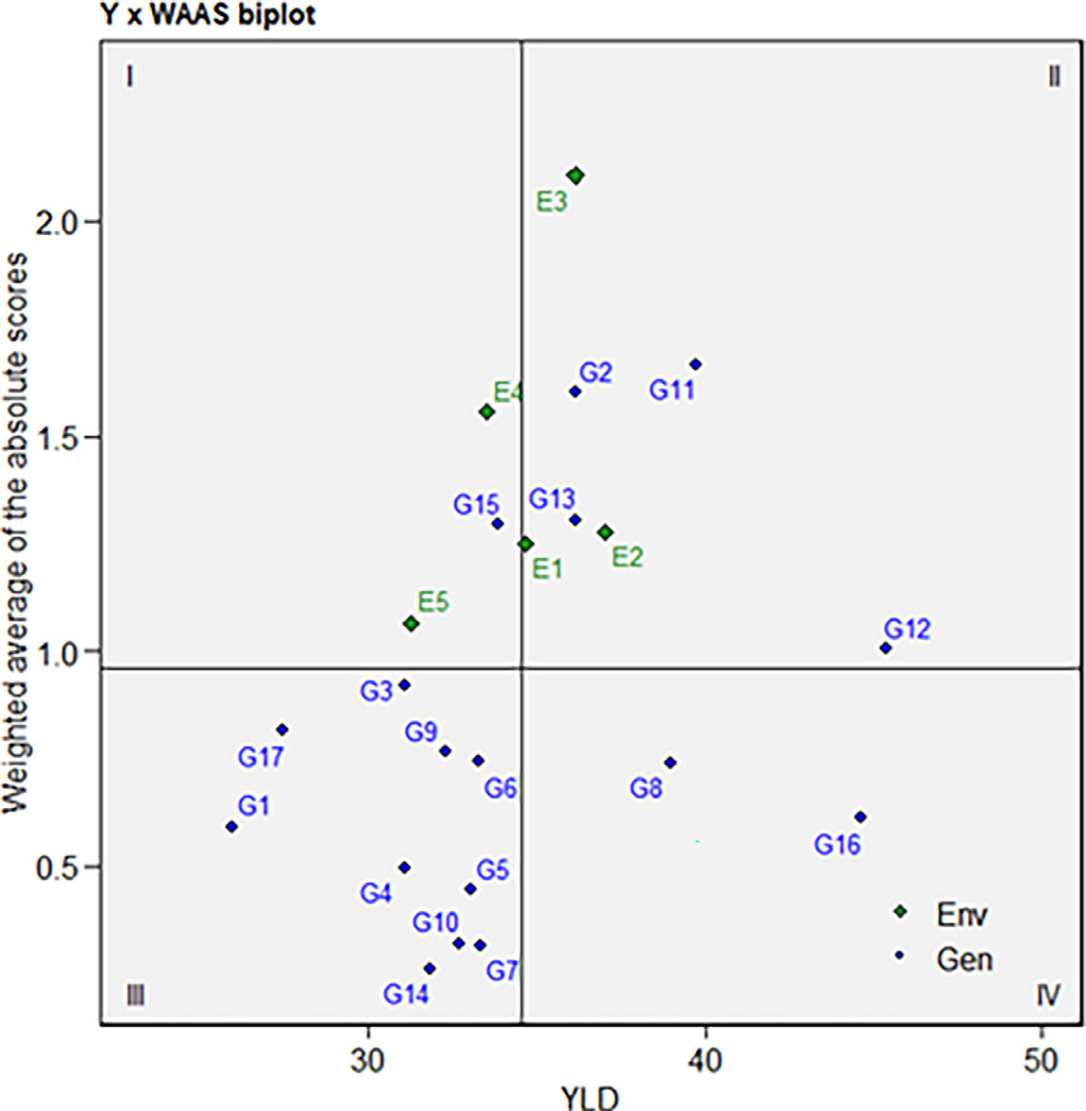
The WAAS biplot of the AMMI model with the mean performance of the root yield for 17 sweet potato genotypes tested across five locations of Bangladesh during the 2022–23 growing season.

Fig. 1 presents a WAAS biplot illustrating the performance of 17 sweet potato genotypes across five locations. The central vertical line indicates the average root yield across all locations, with genotypes positioned to the right showing higher yields and those on the left displaying lower yields. The horizontal axis, situated in the middle of the biplot, represents the mean of WAAS. The biplot is divided into four quadrants based on the intersection of this axis with the vertical axis, allowing for the categorization of genotypes based on their adaptability to different locations. In the WAAS biplot, G15 was positioned in the first quadrant, G12, G13, G2, and G11 in the second quadrant, G3, G17, G9, G6, G1, G4, G5, G10, G7, and G14 in the third quadrant, and G8 and G16 in the fourth quadrant.

**Fig. 2:**
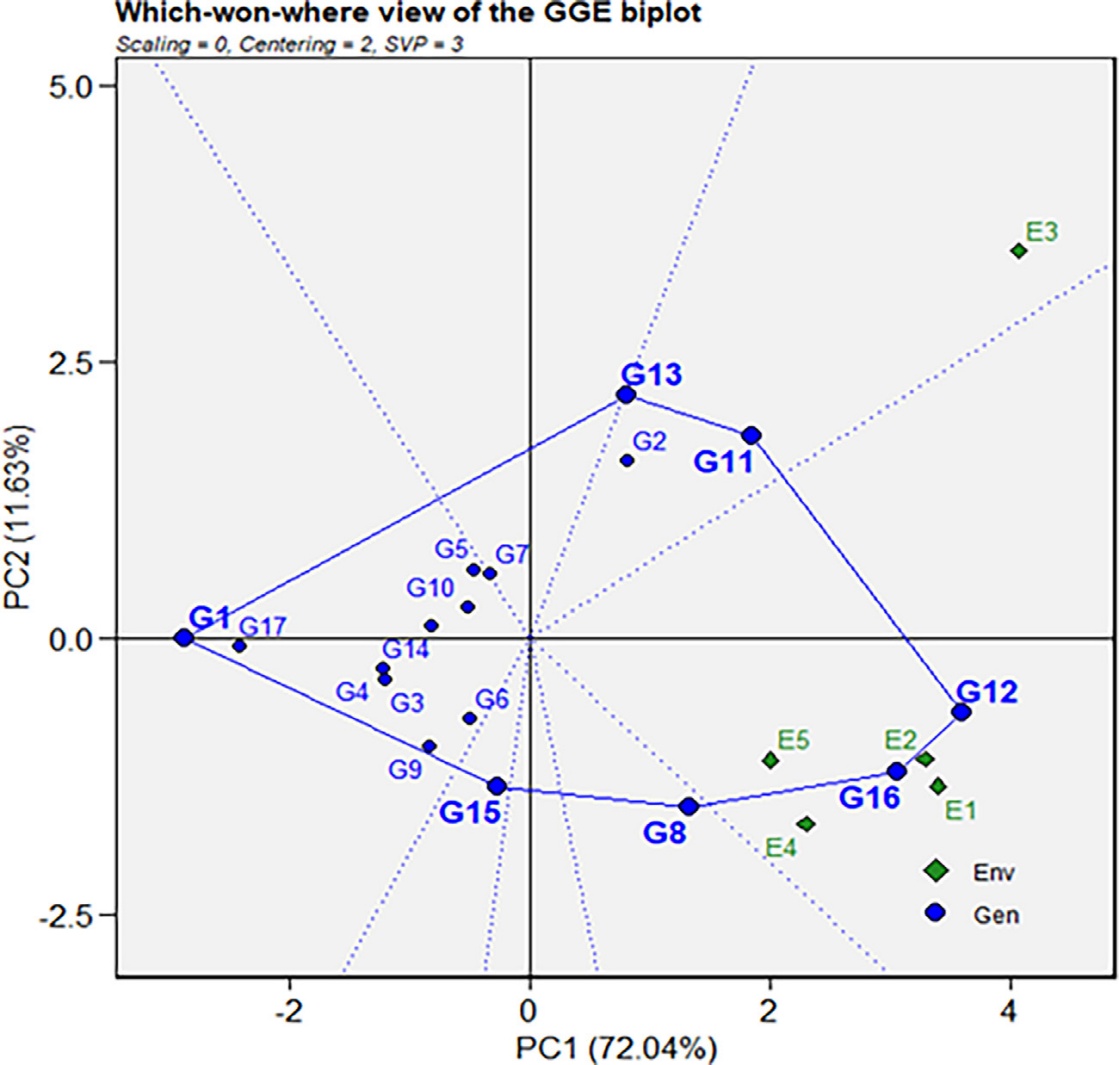
Polygonal view of the GGE biplot for winner genotype for root yield in respective location.

In the polygonal biplot illustrated in Fig. 2, a shape is created by connecting the genotype vertices, namely G12, G16, G8, G15, G1, G13, and G11. This polygon is further divided into seven distinct segments by dotted lines radiating from the plot's origin and extending perpendicular to the polygon's sides. Within one segment, G11 and G2 are associated with location E3, while another segment features G12 and G16 along with locations E1, E2, E4, and E5. The remaining five segments indicate genotypes without any specific location association.

## 4 Experimental Design, Materials and Methods

### 4.1 Descriptions of experimental sites

The research was carried out in the course of the 2022–23 growing season in five different regions of Bangladesh, namely Gazipur, Bogura, Jamalpur, Jashore, and Chattogram. The selection of these locations aimed to encompass a broad range of environmental conditions present in Bangladesh. In order to offer an initial insight into the soil and environmental characteristics of the study area, n Table 1 provides a brief summary of the five selected locations [4,[6], [7], [8]].

### 4.2 Experimental materials

Seventeen different sweet potato varieties obtained from the TCRC, BARI, Bangladesh, were utilized in the study. A comprehensive description of these genotypes can be found in Supplementary Table 2 [2,4,9]. The study employed a RCBD with three replications in each site.

### 4.3 Experimental design, management practices, and data collection

The research utilized a randomized complete block design (RCBD) with three replications at each site. In this design, ten vines of each genotype were planted in a single row, and a single plot consisted of five rows of one genotype. These plots were replicated three times. The guidelines outlined by Alam et al. [4] were followed for preparing the experimental site, implementing management practices, and harvesting the storage roots. Regular inspections and the use of pesticides resulted in minimal disease and insect issues. Harvesting of storage roots occurred 130 days after planting, with ten randomly selected plants from each plot in every replication. The calculation of storage root yield (YLD) involved dividing the average yield of the ten selected plants by the total number of plants in a one-hectare land area.

### 4.4 Statistical analysis

The investigation utilized a combined two-way analysis of variance (ANOVA) and mean separation for examining genotypes (G), location (E), and genotype-location (environment) interactions (GEI). To discern mean values at a significance level of *p* < 0.05, the least significant difference (LSD) test was applied. The research incorporated AMMI analysis of variance. The ``metan'' package in the R statistical analysis system version 4.2.0 [10] was employed for graphical representations, including the WAAS biplot based on the AMMI model and the graphical presentation of the GGE biplot.

## Limitations

Since the dataset encompasses only a single growing season, it may not fully capture the variations observed across different years. Variables such as climate, soil conditions, and pest prevalence, which can influence sweet potato yield, may undergo changes annually.

## Ethics Statement

All authors have read and follow the ethical requirements for publication in Data in Brief and our work meets these requirements. Our work does not involve studies with animals and humans.

## CRediT authorship contribution statement

**Zakaria Alam:** Conceptualization, Methodology, Supervision, Visualization, Writing – original draft, Writing – review & editing. **Sanjida Akter:** Validation, Software. **Md. Anwar Hossain Khan:** Methodology, Investigation, Data curation. **Md. Shamshul Alam:** Methodology, Investigation, Data curation. **Md. Nazmul Islam:** Methodology, Investigation, Data curation. **Abul Fazal Mohammad Shamim Ahsan:** Methodology, Investigation, Data curation. **Mohammad Saidur Rahman:** Methodology, Investigation, Data curation. **Md. Babul Anwar:** Methodology, Investigation, Data curation. **Mousumi Sultana:** Methodology, Investigation, Data curation. **S.M. Kamrul Hasan Chowdhury:** Methodology, Investigation, Data curation. **Mohammad Quamrul Islam Matin:** Methodology, Investigation, Data curation. **A.K.M. Zonayed-Ull-Noor:** Methodology, Investigation, Data curation. **Mohammad Mainuddin Molla:** Methodology, Investigation, Data curation. **Umakanta Sarker:** Writing – review & editing.

## Data Availability

Stability and performance analysis of storage root yield in a dataset of sweet potato varieties (Ipomoea batatas L.) (Original data) (Mendeley Data)

## Appendix Supplementary materials

Image, application 1
