# Supplementary material for: Stability and performance analysis of storage root yield in a dataset of sweet potato varieties (Ipomoea batatas L.)
Source: Data Brief. 2024 May 8;54:110493. doi: 10.1016/j.dib.2024.110493 (PMC11109303; doi:10.1016/j.dib.2024.110493)
Supplement: Supplementary file 1 [file mmc1.docx]

Supplementary Table 1. Description of soil and environmental condition of study areas

| Locations (Codes) | Soil | | | | Weather (October, 2022- March, 2023) | | |
| --- | --- | --- | --- | --- | --- | --- | --- |
|  | Agro-ecological representation | Range of altitude | Soil texture | pH (H_2_O) | Total rainfall (mm) | Avg. night temperature (ºC) | Avg. day temperature (ºC) |
| Gazipur (E1) | AEZ28 | 34 | SCL | 5.73 | 283.08 | 17.25 | 28 |
| Bogura (E2) | AEZ25 | 20 | SL | 6.23 | 163.71 | 17 | 27.75 |
| Jamalpur (E3) | AEZ9 | 24 | SL | 6.48 | 182.87 | 17.75 | 28.5 |
| Jashore (E4) | AEZ18 | 6 | SL | 6.61 | 272.75 | 17.25 | 28.25 |
| Chattogram (E5) | AEZ11 | 29 | STL | 4.58 | 218.67 | 18.75 | 27 |

AEZ28- Madupur Tract, AEZ25-Level Barind Tract, AEZ9-Old Brahmaputra Floodplain, AEZ18-Young Meghna Estuarine Floodplain, AEZ11-High Ganges River Floodplain, SCL-silty clay loam, SL- sandy loam, STL-silty loam

Supplementary Table 2. A detailed description of BARI released seventeen sweet potato varieties used in this study

| Variety | Code | Origin | AL (m) | YR | Pedigree | BM | PH (cm) | RDM (%) | BC (mg/100g) | RS | RSC | RFC | FSC |
| --- | --- | --- | --- | --- | --- | --- | --- | --- | --- | --- | --- | --- | --- |
| BARI Mistialu-1 | G1 | Taiwan | 1150 | 1985 | NA | Introduction | 114.00 | 29.82 | 0.99 | Elliptic | Cream | Pale yellow | Pink |
| BARI Mistialu-2 | G2 | Philippines | 442 | 1985 | NA | Introduction | 138.00 | 21.67 | 13.59 | Elliptic | Red | Dark yellow | Green |
| BARI Mistialu-3 | G3 | Bangladesh | 105 | 1988 | NA | Selection | 123.47 | 28.56 | 0.03 | Long elliptic | White | Pink |  |
| BARI Mistialu-4 | G4 | Bangladesh | 105 | 1994 | NA | Full diallel | 117.87 | 27.97 | 7.23 | Ovate | Pale orange | Dark orange |  |
| BARI Mistialu-5 | G5 | Bangladesh | 105 | 1994 | NA | Full diallel | 119.73 | 22.79 | 4.41 | Oblong | Red | Pale orange |  |
| BARI Mistialu-6 | G6 | Peru | 1555 | 1998 | NA | Introduction | 127.07 | 24.15 | 1.04 | Oblong | Pale orange | Pale orange |  |
| BARI Mistialu-7 | G7 | Peru | 1555 | 1998 | NA | Introduction | 121.60 | 28.99 | 0.69 | Oblong | White | Pale yellow | Pink |
| BARI Mistialu-8 | G8 | Peru | 1555 | 2008 | CIP- 440025 | Introduction | 120.13 | 35.82 | 1.76 | Long irregular | Pink | Pale yellow |  |
| BARI Mistialu-9 | G9 | Peru | 1555 | 2008 | CIP- 440074.2 | Introduction | 96.76 | 20.04 | 4.41 | Long irregular | Red | Light orange |  |
| BARI Mistialu-10 | G10 | Bangladesh | 105 | 2013 | ♀H_8_ | Half diallel | 103.40 | 27.14 | 0.12 | Oblong | White | Dark orange |  |
| BARI Mistialu-11 | G11 | Bangladesh | 105 | 2013 | SP-613 | Selection | 113.33 | 35.70 | 0.04 | Long elliptic | Pink | Cream |  |
| BARI Mistialu-12 | G12 | Peru | 1555 | 2013 | CIP- 440001 | Introduction | 124.13 | 35.93 | 5.46 | Long oblong | Cream | Pale orange |  |
| BARI Mistialu-13 | G13 | Peru | 1555 | 2013 | CIP- 440014 | Introduction | 130.93 | 29.35 | 6.12 | Long oblong | Yellow | Light orange |  |
| BARI Mistialu-14 | G14 | Bangladesh | 105 | 2017 | CIP- 441132 | Introduction | 149.33 | 25.06 | 14.67 | Long irregular | Pink | Dark orange |  |
| BARI Mistialu-15 | G15 | Peru | 1555 | 2017 | CIP- 440267.2 | Introduction | 102.25 | 21.94 | 12.35 | Long irregular | Pink | Dark orange |  |
| BARI Mistialu-16 | G16 | Bangladesh | 105 | 2018 | ♀H_2_/08 | Half diallel | 122.12 | 23.42 | 16.20 | Long irregular | White | Dark orange |  |
| BARI Mistialu-17 | G17 | Indonesia | 367 | 2021 | Antho SP-01 | Introduction | 98.22 | 35.45 | NA | Long irregular | Pink | Pink |  |

AL= altitude of origin, YR= year of release, NA= information not available, BM= methods of breeding, PH= matured plant height, RDM= storage root dry matter, BC= beta carotene content of fresh storage root, RS= storage root shape, RSC= storage root skin color, RFC= storage root flesh color, FSC= fresh stem color
